# Universal Screening in Primary Care Practices by Self-administered Tablet vs Nursing Staff

**DOI:** 10.1001/jamanetworkopen.2022.1480

**Published:** 2022-03-08

**Authors:** David P. Miller, Kristie L. Foley, Richa Bundy, Ajay Dharod, Elena Wright, Mark Dignan, Anna C. Snavely

**Affiliations:** 1 Department of Internal Medicine, Wake Forest School of Medicine, Winston-Salem, North Carolina; 2 Department of Implementation Science, Wake Forest School of Medicine, Winston-Salem, North Carolina; 3 Department of Internal Medicine, College of Medicine, University of Kentucky, Lexington; 4 Department of Biostatistics and Data Science, Wake Forest School of Medicine, Winston-Salem, North Carolina

## Abstract

This nonrandomized controlled trial investigates whether self-administered screening via an in-office tablet app was associated with improved detection of patients at risk for depression, injurious falls, or intimate partner violence compared with screening performed by clinicians.

## Introduction

Screening primary care patients for depression, injurious falls, or intimate partner violence is hampered by time pressures, staff discomfort, and patients’ reluctance to disclose sensitive information.^1,2^ Self-administered screening on a tablet device could address these barriers and improve the detection of at-risk patients.

## Methods

The Wake Forest University Health Sciences institutional review board approved this nonrandomized controlled trial with a waiver of informed patient consent as the research involved no more than minimal risk and could not be practicably carried out without the waiver. We followed the Transparent Reporting of Evaluations With Nonrandomized Designs (TREND) reporting guideline for nonrandomized evaluations except the nature of our intervention prevented blinding (trial protocol in Supplement 1).

Our team created an app, called mPATH, that patients access on a tablet at their primary care office to assist with colorectal cancer screening. To encourage practices to use the app, we included screening questions for depression (Patient Health Questionnaire-2 and -9),^3^ fall risk,^4^ and intimate partner violence that the participating health system requires nursing staff to ask at every visit. All adult patients were given the tablet with the app at check-in, and it transmitted results to the electronic health record. We are currently conducting a cluster randomized implementation-effectiveness study of the app in community-based practices with an anticipated completion date in March 2023 (NCT03843957). For this nonrandomized controlled trial, we evaluated data from the first 6 participating practices (3 family medicine and 3 internal medicine) to determine if more patients with depression, fall risk, or intimate partner violence were identified in the 60 days after the app was launched compared with 60 days before, when nursing staff were asking the same screening questions verbally. We included all patients aged 18 years or older seen during these periods (June 2019 to February 2020). To avoid confounding from COVID-19, we truncated the post period for the last 2 enrolled clinics at 30 days.

The primary outcome was the proportion of patients who screened positive for depression, fall risk, or intimate partner violence, and secondary outcomes included high-risk screening results that might trigger immediate action. All analyses were intention to treat and conducted using R statistical software version 3.6.1 (R Project for Statistical Computing). All models (logistic regression and binomial) controlled for practice; logistic models also included patient demographics. Race and ethnicity were self-reported in the EHR data we abstracted.

## Results

Among 23 026 patients included in the study, 13 342 (57.9%) were female, 18 542 (80.5%) were non-Hispanic White, and 3098 (13.5%) were Black or African American; mean age was 59.7 years (range, 18-102 years). Patient demographics were similar in the periods before (n = 12 239) and after (n = 10 787) implementation (Table 1). Use of the app varied from 10.3% to 60.5% across the 6 clinics, primarily because of differences in how often front desk staff handed the tablet to patients. Despite suboptimal uptake, more than twice as many patients screened positive after the app was launched (Table 2). Significant increases were seen across all clinics and for all screening domains, including for patients who reported thoughts of self-harm, injurious falls, or “that conflicts sometimes turn into physical fights.” The association of the app with the primary outcome was unchanged after accounting for patient characteristics (adjusted odds ratio, 2.6; 95% CI, 2.4-2.8). Sensitivity analyses yielded similar results.

**Table 1.** Characteristics of Patients Seen During Study Time Periods

Characteristic	Patients, No. (%)		
	All patients (n = 23 026)	Time period	
		Before tablet (n = 12 239)	After tablet (n = 10 787)
Sex			
Female	13 342 (57.9)	7030 (57.4)	6312 (58.5)
Male	9684 (42.1)	5209 (42.6)	4475 (41.5)
Age, mean (range), y	59.7 (18-102)	60.2 (18-102)	59.0 (18-102)
Race and ethnicity			
Asian	324 (1.4)	161 (1.3)	163 (1.5)
Black/African American	3098 (13.5)	1640 (13.4)	1458 (13.5)
Hispanic or Latino	745 (3.2)	388 (3.2)	357 (3.3)
White, non-Hispanic	18 542 (80.5)	9876 (80.7)	8666 (80.3)
Other^a^	317 (1.4)	174 (1.4)	143 (1.3)
Primary health insurance			
Commercial	10 344 (44.9)	5382 (44.0)	4962 (46.0)
Medicaid	778 (3.4)	401 (3.3)	377 (3.5)
Medicare	10 522 (45.7)	5705 (47.6)	4817 (44.7)
Uninsured	829 (3.6)	462 (3.8)	367 (3.4)
Other	553 (2.3)	289 (2.4)	264 (2.4)
Practice			
A	3665 (15.9)	1935 (15.8)	1730 (16.0)
B	3442 (14.9)	1574 (12.9)	1868 (17.3)
C	3820 (16.6)	1943 (15.9)	1877 (17.4)
D^b^	4739 (20.6)	2866 (23.4)	1873 (17.4)
E	4385 (19.0)	2215 (18.1)	2170 (20.1)
F^b^	2975 (12.9)	1706 (13.9)	1269 (11.8)

^a^ Other race and ethnicity categories included American Indian or Alaska Native, Hawaiian or Other Pacific Islander, Other, and Unknown.

^b^ The period of observation following implementation at the practice was truncated at 30 days because of the COVID-19 pandemic.

**Table 2.** Proportion of Patients Screening Positive Before and After Self-administered Tablet Screening (N = 23 026)

Outcome	Time period, No. (%)		Adjusted difference^a^ % (95% CI)
	Before tablet (n = 12 239)	After tablet (n = 10 787)	
Positive screen for composite outcome (depression, fall risk, or intimate partner violence)			
Overall	1067 (8.7)	2102 (19.5)	10.3 (9.5-11.2)
By specific practice			
A	224 (11.6)	509 (29.4)	17.9 (15.2-20.5)
B	94 (6.0)	463 (24.8)	18.8 (16.5-21.1)
C	55 (2.8)	235 (12.5)	9.7 (8.0-11.4)
D	165 (5.8)	242 (12.9)	7.2 (5.4-9.0)
E	265 (12.0)	418 (19.3)	7.3 (5.1-9.5)
F	264 (15.5)	235 (18.5)	3.0 (0.2-5.9)
Positive screens in all practices for:			
Depression (PHQ-2 score >2)	188 (1.5)	450 (4.2)	2.8 (2.3-3.2)
At least moderately severe (PHQ-9 > 14)	82 (0.7)	206 (1.9)	1.3 (1.0-1.6)
At least moderately severe and untreated^b^	63 (0.5)	139 (1.3)	0.8 (0.6-1.1)
Severe (PHQ-9 > 19)	28 (0.2)	86 (0.8)	0.6 (0.4-0.8)
Thoughts of self-harm	39 (0.3)	116 (1.1)	0.7 (0.5-1.0)
Fall risk	907 (7.4)	1692 (15.7)	7.9 (7.1-8.7)
Injury during a fall	208 (1.7)	491 (4.6)	2.7 (2.2-3.1)
Intimate partner violence	10 (0.1)	310 (2.9)	2.3 (2.0-2.6)
Conflicts sometimes turn into physical fights	1 (0)	57 (0.5)	0.5 (0.4-0.6)

Abbreviations: PHQ-2, Patient Health Questionnaire-2; PHQ-9, Patient Health Questionnaire-9.

^a^ Overall differences are adjusted to account for clustering by practice using a binomial model with identity link that includes practice as fixed effects. Individual practice results for the composite outcome are unadjusted.

^b^ Untreated defined as no antidepressant in the patient’s current medication list in the electronic health record.

## Discussion

Compared with verbal screening, self-administered screening with a tablet-based app detected more than twice as many patients with concerns that could warrant immediate clinical attention. A strength of our study is its highly pragmatic design that assesses the performance of tablet-based screening in actual practice. Prior studies show that patients feel tablet-based screening is more private than face-to-face screening^5^ and therefore may disclose more sensitive information.^6^ Busy clinical staff also may paraphrase screening items or ask them in a leading fashion. Limitations of our study include the single health system setting with a predominantly White population, the risk of confounding inherent in any nonrandomized study, and the lack of validation of the health system’s intimate partner violence screening questions. We are currently conducting a multisite randomized controlled cluster study to identify effective strategies for incorporating the program into routine care, and results will be forthcoming.

## Notes

Supplement 1.
Trial Protocol

Supplement 2.
Data Sharing Statement
