# Supplementary material for: Universal Screening in Primary Care Practices by Self-administered Tablet vs Nursing Staff
Source: JAMA Netw Open. 2022 Mar 8;5(3):e221480. doi: 10.1001/jamanetworkopen.2022.1480 (PMC8905387; doi:10.1001/jamanetworkopen.2022.1480)
Supplement: Supplement 1. — Trial Protocol [file jamanetwopen-e221480-s001.pdf]

Appendix D – Effect of mPATH on Screening for Depression, Fall Risk, and Safety

Wake Forest policy requires primary care clinics to screen patients aged 18 and older for depression, fall risk, and safety at home at each provider visit. Nursing staff conduct this screening during the rooming process by asking patients standardized questions and recording their responses in the electronic health record.

We designed mPATH to save staff time by having patients answer these same screening items on an iPad and then transmitting their answers to WakeOne. It is possible that mPATH will increase detection of depression, fall risk, and safety concerns because: 1) mPATH systematically asks these screening items of all patients, and 2) patients may feel more comfortable answering these items in an iPad survey than during an in-person interview.

To determine the effect of mPATH on the performance of this screening, we will examine a limited use dataset comparing the 2 months before a clinic started using mPATH to the 2 months after the clinic began using mPATH. Because clinics may need some time to fully adopt mPATH, we will exclude the first two weeks following the launch of mPATH.

Study Population

We will create a limited use dataset containing patients who are seen in one of the first six Wake Forest Baptist Health primary care clinics that are participating in the mPATH Effectiveness and Implementation trial.

<u>Patient inclusion criteria</u>:

- Age 18 or older
- Completed a provider visit at the study clinic during the 60 days prior to the clinic launching the mPATH program (the “pre” time period), or completed a provider visit at the study clinic during days 14 – 73 after the launch of mPATH (the “post” time period)
- Have a preferred language of English or Spanish

<u>Patient exclusion criteria</u>:

- Requiring a language interpreter for a language other than Spanish

Experimental Methods

The limited use dataset will contain the following data elements for each completed patient visit:

- Date of clinic visit (where each date is indicated by a number relative to the clinic’s Launch Date)
- Clinic (designated by a unique study clinic identifier)
- Type of visit completed (for example, new patient visit, return patient visit, annual exam)

- Patient age
- Patient gender
- Patient race/ethnicity
- Patient primary insurance
- Nursing staff who roomed the patient (designated by a unique study identifier)
- Whether patient used mPATH-CheckIn program (Y/N)
- Whether nursing staff used mPATH Nursing Module to transmit mPATH data to WakeOne (Y/N)
- Whether patient has a diagnosis of depression in the problem list in the EHR (Y/N)
- Whether patient has an antidepressant medication listed in the active medication list (Y/N)
- Results of depression screening items
- Results of fall risk screening items
- Results of safety at home screening items

The study dataset will be maintained using the procedures outlined in **Section 8.0 (Confidentiality and Privacy)**. Of note, we are taking the following additional safeguards to further protect patient confidentiality:

1. Dates of clinic visits will be designated using numbers relative to each clinic's launch date, rather than using the actual date
2. Clinics will be designated with a unique study clinic identifier instead of clinic name
3. Nursing staff will be designated with a unique study identifier instead of actual name

Definition of Time Periods

- The day a clinic launches mPATH will be designated as that clinic's Day 0
- The "pre" time period will be Days -60 to -1 (the 60 day period before mPATH Launch)
- The "post" time period will be days 14 to 73 (the 60 day period commencing 2 weeks after mPATH Launch)

Outcome Measures

Primary outcome: The primary outcome will be the proportion of patients seen in each time period ("pre" vs. "post") who screen positive for depression, fall risk, or safety concerns at home.

Secondary outcomes: Secondary outcomes will include the proportion of patients in each time period who:

- Screen positive for depression using the PHQ-2 items. In addition, the outcomes below will be assessed:
    - Have a PHQ9 score >14 (indicating moderately severe depression or severe depression)
    - Have a PHQ9 score >19 (indicating severe depression)
    - Have a PHQ9 score >14 and are not currently taking a medication for depression
    - Have thoughts of self-harm
- Screen positive for fall risk at home. In addition, the outcomes below will be assessed:

    - Have fallen in last 6 months
    - Have had a fall with injury
- Screen positive for safety concerns at home. In addition, the outcome below will be assessed:
    - Report that conflicts turn into fights

Analytic plan
Our primary analysis will compare the proportion of patients seen in the “pre” time period to the “post” time period who screen positive for either depression, fall risk, or safety at home. If a patient is seen more than once in a given time period, only the patient’s first visit in that time period will be included in the analyses. We will estimate the proportion who screen positive for each time period and report along with 95% confidence intervals. A chi-square test will be used to provide an unadjusted comparison between the “pre” and “post” proportions. Logistic regression models will then be used to compare the “pre” and “post” proportions while controlling for clinic and patient demographic variables. We will use the same analytic methods for our secondary outcomes. We will also conduct a sensitivity analysis for our primary outcome where we consider all patients’ visits during a given time period (and not limit analyses to a patient’s first visit in the time period only). For this sensitivity analysis, we will compare the proportion of patients who meet our primary outcome definition at any visit during the “pre” and “post” time periods.

Analytic adjustments due to COVID19 pandemic: Due to the COVID19 pandemic, all practices stopped using mPATH in March 2020, which is prior to the closure of the 60-day “post” time period for clinics #5 and #6 in our sample. Therefore, we will truncate the “post” time period for these two clinics to 30 days. To determine if this truncated time period affected our overall results, we will conduct the following sensitivity analyses: (1) limiting the analyses to only the first 4 clinics to enroll, and (2) limiting data collection to 30-days post- for all 6 clinics.
