# Supplementary material for: Universal Screening in Primary Care Practices by Self-administered Tablet vs Nursing Staff
Source: JAMA Netw Open. 2022 Mar 8;5(3):e221480. doi: 10.1001/jamanetworkopen.2022.1480 (PMC8905387; doi:10.1001/jamanetworkopen.2022.1480)
Supplement: Supplement 2. — Data Sharing Statement [file jamanetwopen-e221480-s002.pdf]

Data Sharing Statement

Miller DP, Foley K, Bundy R, et al. Universal Screening in Primary Care Practices by Self-Administered Tablet vs Nursing Staff. *JAMA Netw Open*. 2022;5(3):e221480. doi:10.1001/jamanetworkopen.2022.1480

Data
Data available: No

Additional Information
Explanation for why data not available: This is a highly pragmatic study in which our intervention was delivered as standard of care and we had a waiver of informed consent. We are analyzing data from the participating health system's electronic health record. Because this is routinely collected clinical data, we do not feel it would be appropriate to share with others (patients had not agreed to have their de-identified data shared with others outside the health system).
